# Lubrication Challenges in Deep-Sea Gear Trans-Missions: A Review of High-Pressure and Low-Temperature Effects

**DOI:** 10.3390/ma19051020

**Published:** 2026-03-06

**Authors:** Weiqiang Zou, Xigui Wang, Yongmei Wang, Jiafu Ruan

**Affiliations:** 1 School of Mechatronics and Automation, Huaqiao University, No. 668 Jimei Avenue, Jimei District, Xiamen 361021, China; zhaoxzhit@163.com (W.Z.); rjfnefu1993@126.com (J.R.); 2 School of Motorcar Engineering, Heilongjiang Institute of Technology, No. 999 Hongqidajie Road, Daowai District, Harbin 150036, China; wyr20091207@163.com

**Keywords:** gear transmission system, deep-sea environment, multi-physics coupling modeling, meshing interface texturing, elasto-hydrodynamic lubrication

## Abstract

Deep-sea gear transmission systems encounter critical lubrication challenges arising from the synergistic coupling of extreme hydrostatic pressure and cryogenic temperatures. These environmental stressors induce exponential viscosity escalation in lubricants, precipitating severe fluidity degradation, elevated startup resistance, and lubrication starvation. Concurrently, seawater intrusion triggers lubricant emulsification, additive deactivation, and electrochemical corrosion at meshing interfaces, collectively escalating the risk of catastrophic lubrication failure and compromising long-term operational reliability. This study systematically elucidates the lubrication degradation mechanisms inherent to deep-sea environments and proposes targeted mitigation strategies. Through comprehensive characterization of deep-sea environmental parameters and their impact on lubricant rheological behavior, we critically evaluate the applicability and inherent limitations of conventional Thermal Elasto-Hydrodynamic Lubrication (TEHL) theory under extreme conditions. Our analysis reveals that established TEHL frameworks necessitate substantial modification to accurately capture pressure-viscosity-temperature coupling phenomena and seawater contamination kinetics. Meshing interface texturing, as an effective anti-friction and wear-mitigation strategy, is investigated to delineate its mechanistic pathways for enhancing lubricant film formation and tribological performance under starved lubrication regimes. Key findings demonstrate that optimized micro-texture architectures can effectively compensate for viscosity-induced fluidity deficits and attenuate the deleterious effects of seawater ingress. Critical knowledge gaps are identified, and future research trajectories are charted: (i) multiphysics coupling models integrating thermo-hydrodynamic, chemo-physical, and mechanical degradation processes; (ii) synergistic texture-coating design paradigms; (iii) high-pressure low-temperature experimental validation protocols; and (iv) engineering implementation frameworks for deep-sea gear transmission systems. This review establishes theoretical foundations and provides technical guidelines for robust lubrication design and long-term operational stability of deep-sea transmission equipment.

## 1. Introduction

**Figure 1:**
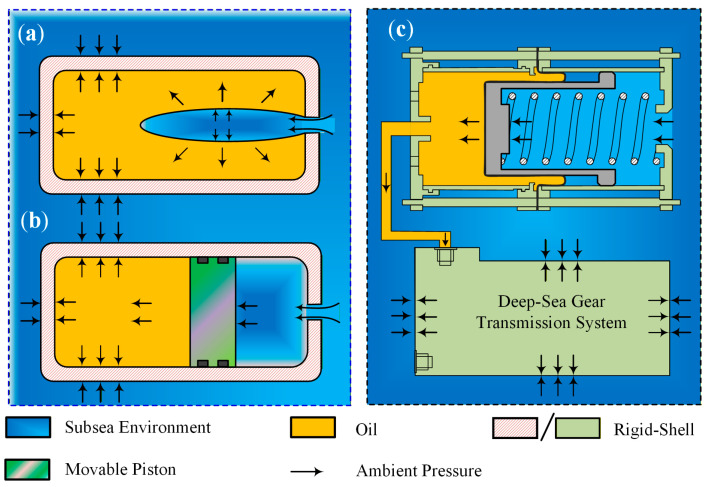
Schematic illustration of the mechanism of pressure-balanced units: (**a**) resilience-bladder type, (**b**) movable-piston type, (**c**) gear transmission system schematic with a rolling diaphragm pressure compensator. (Figure 1a,b: Adapted from Ref. [11]).

The ocean harbors abundant biological, mineral, and energy resources, representing a strategic frontier for sustainable development and a vital space for future human progress [1]. The exploration and utilization of these distant and deep-sea resources rely on advanced equipment such as submersibles, seabed drilling systems, underwater robots, and tidal energy generators [2,3,4]. Within these sophisticated systems, gear transmissions play a pivotal role in power transmission and motion conversion; their performance directly dictates the operational capability, precision, and reliability of the entire equipment [5,6,7]. Unlike terrestrial systems, this equipment is typically deployed at ocean depths ranging from hundreds to thousands of meters, where it is subjected to extreme hydrostatic pressures, near-freezing temperatures, and a highly corrosive saline environment [8,9]. This coupled high-pressure and low-temperature setting poses unprecedented challenges to materials, structures, and lubrication. As equipment descends into deeper waters, the pressure differential across the gearbox increases dramatically. If uncontrolled, the immense external pressure can deform the housing structure and compromise sealing systems, leading to seawater intrusion [10]. To mitigate the immense pressure differentials in deep-sea environments, systems typically employ pressure-balancing devices to maintain pressure equilibrium within the gearbox [11]. Figure 1 illustrates the standard configurations for pressure compensation, including the elastic bladder type (see Figure 1a) and the moving piston type (see Figure 1b). Figure 1c presents a schematic diagram of a gear transmission system incorporating a rolling diaphragm pressure compensator [12]. Although this strategy protects the housing from deformation, it exposes the internal components to high ambient pressure, altering the lubricant’s physicochemical properties. Specifically, high pressure significantly increases oil viscosity and flow resistance, potentially causing additive instability or changes in solubility. This inevitably impairs lubricant film formation and load-bearing capacity, accelerates wear, and reduces the equipment’s operational lifespan. This study addresses the typical deep-sea operating conditions corresponding to the research subject. Following internationally accepted standards in the deep-sea scientific community, the extreme high-pressure environment is specifically defined as 20–110 MPa (corresponding to water depths of 2000–11,000 m), while the low-temperature regime is defined as 1–4 °C (representative of general deep-sea environments excluding hydrothermal vent regions).

Under high-pressure conditions, the viscosity of lubricating oil increases exponentially, leading to a sharp decline in fluidity that can induce a semi-solid state [13,14]. This not only significantly increases churning power loss within the gearbox but also impedes timely backflow and oil replenishment, resulting in film rupture and exacerbated meshing interface wear [15,16]. The high-pressure environment places stringent demands on sealing systems; seal failure can lead to high-pressure seawater intrusion, contaminating the lubricant, corroding gear meshing interfaces, and potentially triggering complex failures such as fretting corrosion [17,18]. Temperatures in most deep-sea regions remain between 2 and 4 °C year-round. These low temperatures further increase viscosity and reduce fluidity [19]. Under the combined effects of high pressure and low temperature, the dramatic rise in viscosity leads to higher starting torque and reduced pumping performance, making it challenging to supply lubricant to the meshing zone promptly and thereby causing lubrication starvation [20]. Moreover, high pressure and low temperature alter critical thermophysical parameters, such as density and thermal conductivity, compromising the accuracy of film thickness and temperature field calculations in Thermal Elasto-Hydrodynamic Lubrication (TEHL) analysis. In extreme cases, such as during cold starts, the drastic viscosity increase may prevent startup altogether [21,22]. As the core theory for studying lubrication in high-load, non-conformal contacts, TEHL comprehensively accounts for hydrodynamic effects, elastic deformation, and the lubricant’s pressure-viscosity and temperature-viscosity characteristics, alongside thermal effects. It accurately describes key parameters in the meshing zone, such as film pressure, thickness, temperature field, and friction [23,24,25]. By establishing numerical models that reflect the coupled effects of high pressure and low temperature, applying TEHL theory to deep-sea gear systems enables the quantitative analysis and prediction of lubrication characteristics under extreme operating conditions [26]. However, relying solely on passive selection of superior lubricants and base materials is often insufficient to address such harsh conditions fully. Interface texturing technology, an active tribological control strategy, has garnered significant attention in recent years [27]. This technique involves creating micro-scale pits or grooves with specific geometries and spatial arrangements on friction pair interfaces to improve lubrication and suppress friction and wear. Through mechanisms such as acting as oil reservoirs, generating secondary hydrodynamic effects, and trapping wear debris, interface texturing offers a promising solution for addressing the lubrication challenges faced by deep-sea gears under high loads and low speeds.

While traditional research on gear lubrication has achieved substantial success under conventional conditions, it remains inadequate for addressing extreme marine environments characterized by high pressure and low temperatures. Adequate lubrication is essential for reducing friction and wear, lowering energy consumption, and extending equipment service life; however, traditional lubrication theories and methods often prove insufficient in deep-sea, high-pressure, and low-temperature environments. This review critically evaluates the progress of two core research areas, Elasto-Hydrodynamic Lubrication (EHL) theory and interface texturing technology, within the context of marine gear transmission systems. Through systematic analysis of lubrication performance degradation mechanisms under extreme operating conditions, prospective research directions are delineated. This study aims to provide theoretical support for the reliable design and operation of marine transmission systems, offering valuable insights and engineering guidance for enhancing the performance and reliability of high-end deep-sea gear equipment.

## 2. Lubricant Interactions in Deep-Sea Extremes: A Review of Environmental Characteristics and System Responses

### 2.1. Multi-Scale Parametric Characteristics of Deep-Sea Environments: Pressure and Temperature

In contrast to terrestrial operating environments, marine gear systems operate in a significantly more severe service environment, compounded by high pressure and low temperatures. As operational depths reach several thousand meters, systems must adapt to these extreme conditions. The deep sea, commonly defined as depths exceeding 200 m or, more strictly, below 1000 m, constitutes over 95% of the Earth’s habitable biosphere and represents the planet’s most extensive and least understood ecosystem [28]. Seawater temperature drops noticeably with depth, stabilizing only after passing through the thermocline into the abyssal and hadal zones [29]. Beginning at approximately 200 m depth, seasonal and interannual temperature fluctuations essentially vanish, creating a highly stable cold environment throughout the global deep ocean where water temperatures are consistently maintained between 0 and 4 °C [30,31]. Concurrently, hydrostatic pressure increases sharply, rising linearly at a gradient of about 0.1 MPa per 10 m, exceeding 110 MPa at the extreme depths of the Mariana Trench [32,33]. Light intensity decays exponentially with depth, with 99% of incident light dissipated by 150 m, rendering the environment below 250 m virtually devoid of light [34,35]. Chemically, seawater acts as a strong electrolyte, rich in chloride ions and dissolved oxygen, and constitutes a naturally corrosive environment [36]. In this aqueous setting, metals form microscopic anodic and cathodic regions where anodic metal dissolution and cathodic oxygen reduction reactions occur. Notably, the high concentration of chloride ions is highly aggressive and can break down the passivation film on metal interfaces, thereby initiating and accelerating localized corrosion [37]. Prolonged exposure to this high-salinity, high-humidity environment poses severe electrochemical corrosion challenges for metallic materials. This not only significantly compromises the load-bearing capacity of materials but also contributes to the premature failure of gear transmission systems.

### 2.2. Thermodynamic and Rheological Responses of Lubricants to Deep-Sea Pressure-Temperature

At low temperatures, the thermal motion of molecules is suppressed, allowing intermolecular forces to dominate and molecular structures to become more ordered. This phenomenon increases flow resistance and elevates viscosity. As temperature rises, however, intermolecular separation expands and cohesive forces decline; the resulting enhancement in molecular thermal motion reduces flow resistance, manifesting macroscopically as a substantial decrease in viscosity [38,39]. On the other hand, increasing pressure compresses lubricant molecules, reducing the average intermolecular distance and free volume. These intensified interactions lead to a marked increase in flow resistance and an exponential increase in viscosity [40]. Within the framework of EHL theory, lubricant viscosity is commonly defined as a function of both pressure and temperature.

Since the 19th century, researchers have developed various empirical and semi-empirical models to characterize the dependence of lubricant viscosity on temperature and pressure. These models generally fall into three categories: viscosity-temperature equations, viscosity-pressure equations, and coupled viscosity-temperature-pressure equations that account for both factors simultaneously. Among viscosity-temperature models, the Reynolds equation is suitable for narrow temperature ranges, whereas the Andrade equation, despite its simple form, has limited predictive capability over broad temperature spans [41,42,43]. The Slotte equation more accurately describes viscosity changes across wide temperature variations, although its parameters are fluid-specific [44]. The Vogel-Fulcher-Tammann (VTF) equation, grounded in free volume theory, is particularly effective for describing viscosity behavior near the glass transition [45]. The Walther equation, recommended as an ASTM standard, serves as the basis for generating viscosity-temperature charts. Regarding viscosity-pressure relationships, the Barus equation is simple but applicable only to low pressures; the Roelands equation offers superior accuracy over a wider pressure range, while the Cameron equation can be viewed as a modification of the Barus model suited for high-pressure conditions [46,47]. In practical operating scenarios, such as EHL, temperature and pressure often fluctuate drastically and simultaneously, necessitating the use of coupled models. For instance, the Barus-Reynolds combination is straightforward but inherits the high-pressure inaccuracies of the Barus model. Since its introduction in 1966, the Roelands equation has gained widespread recognition for its accuracy and universality across diverse conditions. To provide a unified description of viscosity behavior from the liquid to the glassy state, researchers have also developed comprehensive models such as the WLF-Yasutomi model. By integrating the WLF equation with the Tait equation of state, this model effectively predicts viscosity variations across a range of pressures and temperatures within a single framework. Continuously refined through experimental fitting, it has become one of the most representative models in current use [48,49,50].

Under high-pressure operating conditions typical of deep-sea environments, hydrostatic pressures reaching tens of megapascals are sufficient to induce a significant increase in the base viscosity of lubricating oil. The lubricant-related parameters for the deep-sea gear transmission system are listed in Table 1. In Hertzian contact zones, pressures can surge to the gigapascal (GPa) range, increasing viscosity by several orders of magnitude. Accurately modeling such drastic variations is critical for TEHL analysis. The low-temperature environment of the deep sea can elevate lubricant viscosity by one to two orders of magnitude compared to ambient conditions. While increased viscosity theoretically facilitates the formation of thicker oil films, the adverse effects of severely reduced fluidity are more pronounced and often constitute the primary cause of lubrication failure. The synergistic effect of high pressure and low temperature drives the lubricating oil into an extremely high-viscosity state, where its behavior approximates that of an amorphous solid, exhibiting high internal friction and shear stress. This state further acts as a significant source of churning losses and temperature escalation within the contact zone. Consequently, precise characterization and simulation of the viscosity-pressure-temperature relationship under these coupled high-pressure and low-temperature conditions are crucial prerequisites for reliable lubrication analysis and design. However, the experimental data under such conditions remain extremely scarce.

### 2.3. Lubricant Density: A Foundation for Thermophysical Property Estimation and Performance Prediction

Beyond viscosity, thermophysical properties such as density, thermal conductivity, and specific heat capacity are pivotal in TEHL analysis because they directly affect the solution of the mass conservation and energy equations. These properties depend significantly on temperature and pressure, a sensitivity that intensifies under extreme operating conditions. Density, a key parameter, substantially affects the fluid mass flowing through the contact zone and the oil film thickness. Research indicates that density typically decreases with rising temperature and increases with rising pressure [51]. Owing to its concise form and ease of application, the Dowson-Higginson equation is widely used in engineering calculations:

(12) $$\rho \left(p,T\right)=\rho_{R}\left\{\frac{5.9\times 10^{8}+1.34p}{5.9\times 10^{8}+p}-\beta_{\mathrm{DH}}\left(T-T_{R}\right)\right\}$$

where $\rho_{R}$ is the reference density, $T_{R}$ is the reference temperature, and $\beta_{\mathrm{DH}}$ is the density-temperature coefficient.

To further enhance prediction accuracy across a wide range of pressures and temperatures, the Tait [52] equation of state is more precise than the Dowson–Higginson equation:

(13) $$\rho \left(p,T\right)=\frac{\rho_{R}}{\left\{1+\alpha_{\upsilon }\left(T-T_{R}\right)\right\}\left\{1-\frac{1}{1+K_{0}^{\prime }}\ln\left[1+\frac{\left(1+K_{0}^{\prime }\right)p}{K_{\infty }e^{-\beta_{K}T}}\right]\right\}}$$

where $\alpha_{\upsilon }$ is the coefficient of volumetric thermal expansion, $K_{0}^{\prime }$ is the variation in the isothermal bulk modulus at absolute zero temperature, $K_{\infty }$ is the isothermal bulk modulus at absolute zero temperature, and $\beta_{K}$ is the temperature coefficient of the isothermal bulk modulus.

**Figure 2:**
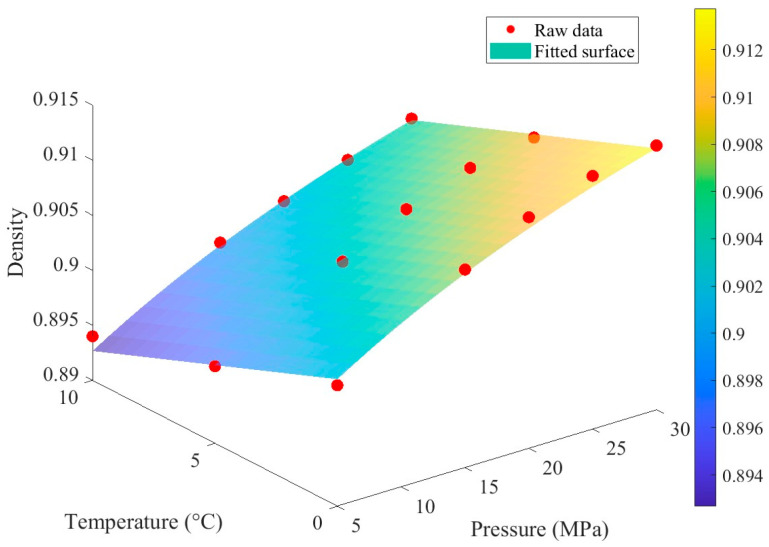
The density variation with respect to pressure and temperature.

**Figure 3:**
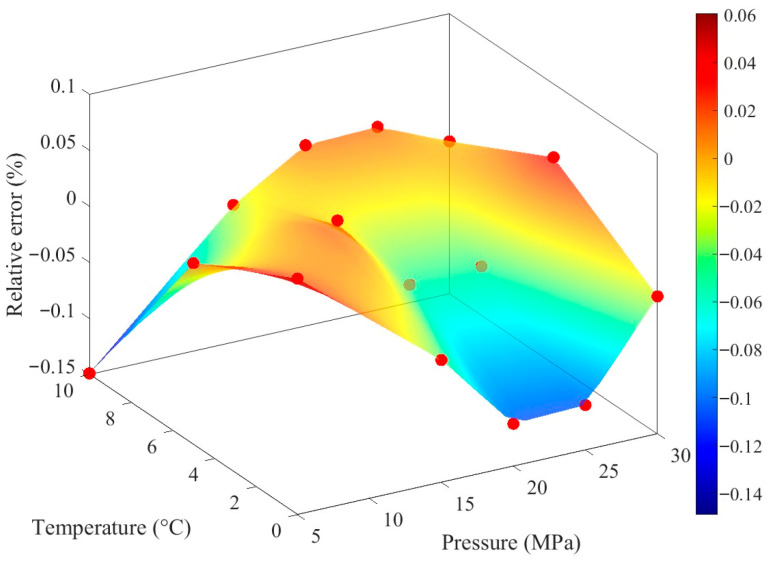
Relative error meshing interface of density. Herein, the red dots in the figure are described as raw data (as indicated in Figure 2).

Thermal conductivity and specific heat capacity define a lubricant’s capacity for heat transfer and thermal storage, respectively. Together, these parameters govern the temperature profile within the TEHL contact zone. While both properties exhibit pressure and temperature dependence, the sensitivity of their variation is generally less pronounced than that of viscosity and density. Typically, thermal conductivity increases slightly with increasing pressure and temperature, whereas specific heat capacity is primarily temperature-dependent, with pressure playing a negligible role. In low-temperature environments, reduced thermal conductivity can impede the dissipation of shear-induced heat, leading to additional localized temperature spikes. This phenomenon may initiate a complex coupled feedback loop characterized by rising temperatures, decreased viscosity, and consequent variations in shear heat generation. Low temperatures and high pressures in deep-sea environments significantly affect the viscosity of lubricating oils, as illustrated in Figure 2 and Figure 3. Low temperatures markedly suppress molecular thermal motion and enhance intermolecular forces, resulting in an exponential increase in viscosity. High pressures further intensify this rise by reducing molecular spacing, potentially leading to non-Newtonian fluid behavior or even solid–liquid phase transitions. When both factors act synergistically, the combined effect of low temperature and high pressure severely impairs the fluidity and film-forming capacity of lubricating oils, causing difficulties in equipment startup or a sharp increase in friction and wear. Based on the stringent demands of marine environments on the temperature and pressure resistance of lubrication systems, a systematic analysis of the physicochemical evolution of lubricating oils under coupled temperature-pressure conditions is urgently required.

### 2.4. Seawater Ingress-Induced Multiscale Degradation of Deep-Sea Gearbox Lubricant: Damage Mechanisms, Performance Prediction, and Intrusion-Resistant Technology

**Figure 4:**
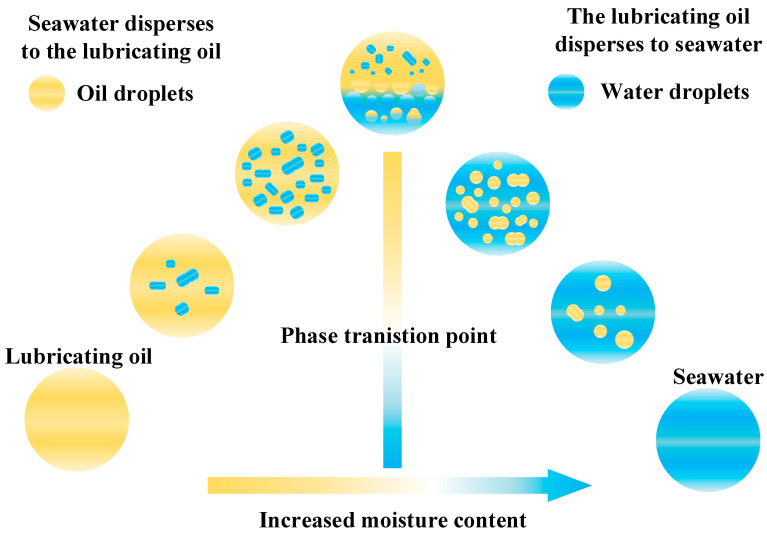
Schematic diagram of oil-water two-phase state [55].

Sealing systems represent a critical weak point in deep-sea equipment. Subjected to sustained high differential pressures, they face a significant risk of failure. If seawater infiltrates the deep-sea transmission system, it drastically reduces the lubricant’s apparent viscosity, impairing film formation and shifting from hydrodynamic to mixed or boundary lubrication, thereby accelerating wear on friction pairs [53]. As illustrated in Figure 4, the phase behavior of oil-water mixtures is complex. Below the saturation limit, water dissolves into the oil to yield a homogeneous solution with reduced viscosity relative to the pure base oil [54]. Beyond this limit, an unstable two-phase system develops, characterized by water dispersed as fine droplets. While initial water addition slightly increases viscosity due to heightened interfacial tension [55], exceeding a critical concentration triggers an emulsion inversion from water-in-oil (W/O) to oil-in-water (O/W). This results in a precipitous decline in viscosity and a consequent loss of the lubricating film’s load-bearing capacity. Electrolytes and chloride ions inherent in seawater can disrupt the oil-water interfacial tension and compromise the metal’s passive film. The synergistic effect of mechanical wear and electrochemical corrosion significantly accelerates material degradation, fostering a vicious cycle of corrosion-promoting-wear and wear-promoting-corrosion [56,57,58].

Moreover, seawater can induce hydrolysis or chemical reactions with extreme pressure and anti-wear additives, thereby neutralizing their efficacy and inhibiting protective film formation under boundary lubrication regimes [59]. Seawater also disrupts the suspension stability of additives and accelerates the hydrolytic degradation of ester-based environmentally friendly lubricants. The resulting production of acidic species and sludge intensifies the corrosion of metal components [60]. This deterioration not only diminishes lubrication performance but also risks clogging filters and oil passages, potentially leading to oil starvation and threatening the integrity of the lubrication system.

**Figure 5:**
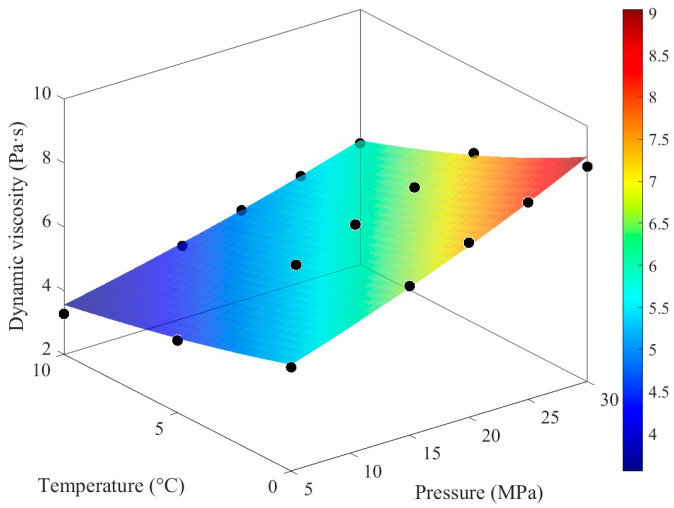
VG320 lubricant Roelands Equation fitting. The black dots in the figure are described as raw data (as indicated in Figure 2).

The fitting results of the Roelands model demonstrate high consistency with experimental data in terms of pressure and temperature trends. The constructed response interface effectively captures the overall distribution of the experimental data points, intuitively illustrating the variation in viscosity under different operating conditions (Figure 5). Error analysis indicates that the residual sum of squares is 0.3926, with an average relative error of 2.29% and a maximum relative error of 8.78%. The low average error reflects the overall reliability of the model fitting, while the relatively high maximum error suggests some deviations under specific conditions.

**Figure 6:**
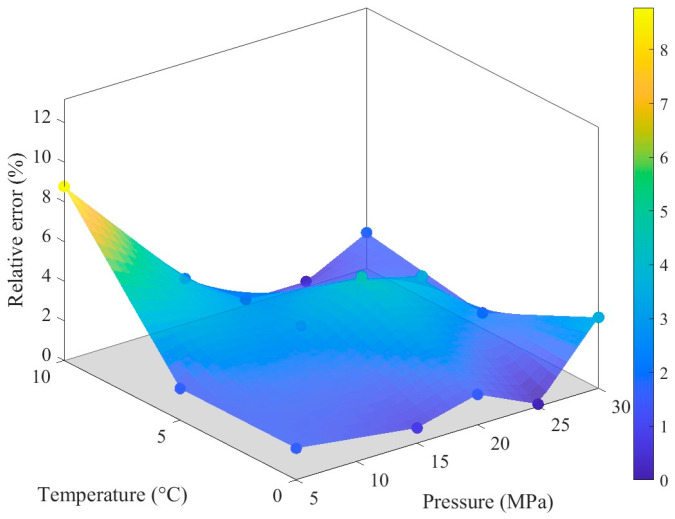
Fitting relative distribution changes. The dots of different colors in the figure represent the raw data (as indicated in Figure 2).

Figure 6 further illustrates the distribution of relative errors across the pressure-temperature parameter space: most data points exhibit small errors, with significant deviations observed only in a few cases. This distribution pattern aligns with the aforementioned statistical results. The variation in lubricant viscosity with temperature and pressure constitutes a fundamental issue in lubrication engineering. The viscosity-temperature relationship characterizes the decrease in viscosity as temperature increases. In this study, the Roelands viscosity-pressure-temperature correlation is fitted using MATLAB R2025a to obtain the dynamic viscosity of the lubricant under various temperature and pressure conditions, as presented in Table 2.

## 3. EHL Theory in Ultra-Deep Marine Conditions: Adaptive Mechanisms, Key Challenges, and Frontiers

### 3.1. Multi-Physics Coupling Effects on Lubrication Regime Transitions in Deep-Sea Gear Transmission Meshing Interfaces

**Figure 7:**
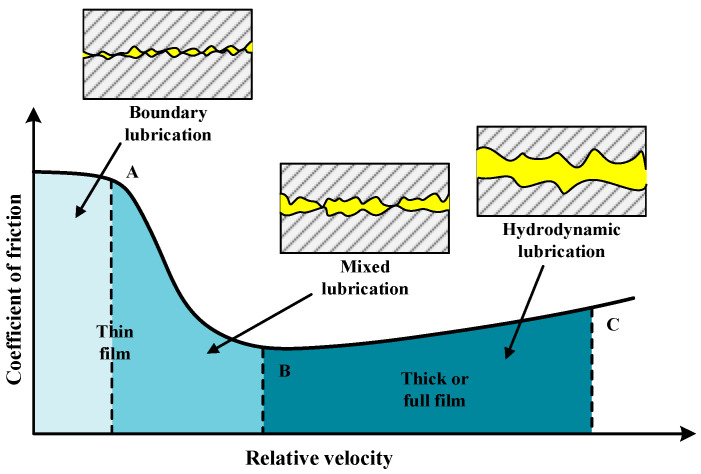
Transition of Lubrication Regimes. A-Boundary lubrication; B-Mixed lubrication; C-Hydrodynamic lubrication.

The Stribeck curve delineates three distinct lubrication regimes for mechanical contacts: full-film, mixed, and boundary lubrication. Full-film lubrication principally comprises hydrodynamic lubrication and EHL. In the context of heavily loaded gear pairs, the operating regime typically spans the spectrum from EHL to mixed lubrication [61]. Figure 7 schematically delineates the characteristic features of these regimes and their transition dynamics. Under hydrodynamic and elastohydrodynamic conditions, contacting interfaces are entirely separated by a lubricant film of adequate thickness. Friction primarily arises from the internal shearing of lubricant molecules.

In this state, the coefficient of friction is markedly low, wear is negligible, and the system operates under ideal lubrication conditions. Boundary lubrication stands in stark contrast; here, the lubricant film is extremely thin or non-existent, and the load is borne almost entirely by direct asperity contact. This regime is characterized by high friction coefficients and severe wear. Mixed lubrication represents the intermediate state, where the load is distributed between the fluid film and asperity contacts, resulting in complex tribological behavior. Under the high-pressure, low-temperature conditions unique to deep-sea environments, lubricant viscosity increases exponentially, profoundly influencing the lubrication regime. Within the full-film regime, increased viscosity promotes the formation of a thicker lubricant film, thereby bolstering interface protection. In the mixed regime, higher viscosity mitigates direct asperity contact, reducing wear. Conversely, in the boundary regime, protection relies primarily on meshing interface-adsorbed chemical films. Nevertheless, the extreme viscosity elevation in such severe environments complicates the lubricant’s rheological behavior, posing a potential threat to the deep-sea gear transmission system’s operational reliability.

High hydrostatic pressure in the deep sea can induce the glass transition of lubricants, leading to a sharp increase in the molecular relaxation time and a shift in dynamic behavior from liquid-like to solid-like, thereby forming a macroscopic friction plateau [62,63]. At low temperatures, lubricants exhibit diverse phase behaviors: mineral oils may exhibit shear-thinning and yield stress driven by paraffin crystallization, whereas synthetic oils may undergo a glass transition, leading to a nonlinear increase in viscosity [64]. These transitions significantly compromise the spreading capability of the oil film, expediting the shift from EHL to mixed or even boundary lubrication regimes [65,66]. Models of rough interface contact further validate that increased interface roughness or reduced load shortens the window for the onset of mixed lubrication; notably, wear frequently initiates at the point of minimum film thickness along the contact zone periphery [67]. In light of the limitations of conventional lubricants in marine settings, solid lubricants suitable for extreme temperatures have emerged as a critical solution. This category encompasses layered materials, soft metals, MAX phases, and stable fluorides and oxides. Self-lubricating composites manufactured via powder metallurgy or thermal spraying techniques provide adequate lubrication across a broad temperature range [68]. Simultaneously, environmentally friendly gel lubricants retain advantageous rheological properties at low temperatures, showing promise for use in polar and deep-sea applications [69].

### 3.2. TEHL Theory for Gears Operating Under High-Pressure and Low-Temperature Conditions

**Figure 8:**
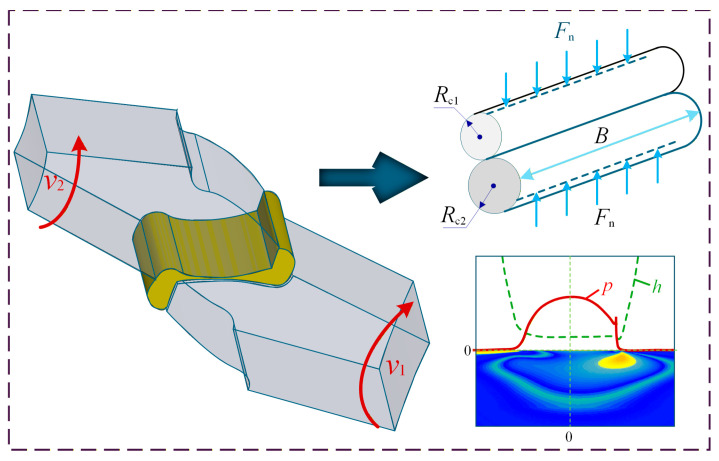
Scheme of pressure distribution and film thickness variation in the lubricating film. Where, the different colors represent the evolution of the lubricating contact film pressure field; symbols, as indicated, represent changes in load, pressure, rotational speed, and film thickness; arrows indicate the direction of rotation and load distribution.

The theory of TEHL for gears comprehensively accounts for the coupling effects of elastic deformation, thermal phenomena, and hydrodynamic lubrication during meshing. This approach enables a more realistic prediction of oil film thickness, pressure distribution, and temperature within the contact zone, thereby facilitating the assessment of performance indicators such as friction, wear, and fatigue [70]. Building upon isothermal EHL theory, TEHL introduces the energy equation to account for frictional heating. The formulation centers on solving a system of highly nonlinear partial differential equations that characterize the physical state of the lubricated contact. A comprehensive TEHL numerical model generally comprises the Reynolds equation, the film thickness equation, the load balance equation, the energy equation, and rheology equations governing the viscosity-pressure-temperature relationship. Numerical techniques are utilized to determine the pressure, thickness, and temperature distributions of the lubricant film. Figure 8 presents a schematic diagram of the pressure distribution and the variation in film thickness of gear meshing interfaces. The lubrication regime of gear meshing falls within the EHL domain, requiring the concurrent consideration of elastic deformation in the contacting bodies and variations in lubricant viscosity.

During actual operation, gear meshing interfaces endure extreme contact pressures, combined rolling and sliding motions, and transient thermal effects, creating a complex TEHL scenario. TEHL theory comprehensively accounts for the coupling among thermal effects, elastic deformation, and hydrodynamics, providing a realistic representation of the lubrication state. Under high-pressure and high-speed transmission, transient temperature elevations in the contact zone further intensify this complexity. Liu et al. [71] elucidated the mechanism by which unbalanced radial forces in high-pressure internal gear pumps induce gear deformation, thereby shifting the dominant frequency of pressure pulsation and integrating dynamic loads with a mixed EHL model. Xiao et al. [72] clarified the evolutionary patterns of gear lubrication parameters under high loads and speeds. Nevertheless, extreme operating conditions present severe challenges to lubrication stability. Duan et al. [73] found that, upon reaching critical load and speed values, the friction coefficient increases sharply when oil temperature exceeds the flash point, precipitating lubrication failure and severe wear. The oil film stiffness model established by Yin et al. [74] demonstrated that thermal-induced temperature rise reduces lubricant viscosity and increases compressive deformation of the oil film, resulting in tangential stiffness significantly higher than that predicted by isothermal assumptions. This phenomenon is particularly pronounced in the tooth root region, where loads are concentrated. Wang et al. [75] developed a finite-element coupling model to investigate mixed lubrication in planetary gear systems under low-speed, heavy-load conditions, accounting for local temperature rises from asperity contact, film rupture, and thermal deformation.

In contrast, low-temperature environments significantly impact lubricant properties and system behavior. Research by Su et al. [76] suggests that low temperatures drastically increase lubricant viscosity and alter its rheology. This results not only in oil film thickening and abnormal fluctuations in the traction coefficient, but also in exacerbated rolling-element slippage. To accurately capture rheological behavior under low-temperature, high-viscosity conditions, Gao et al. proposed a modified Herschel–Bulkley model, which exhibits superior accuracy in fitting experimental data and calculating engineering traction coefficients compared to traditional H-B and T-J models. A mechanical-thermal coupling model by Liu et al. [77] revealed that increased grease viscosity at low temperatures in high-speed train bearings leads to film thickening and aggravated slippage, identifying these factors as intrinsic causes of early failures in cold-region rail bearings. Lastly, a viscous-hyperelastic mixed lubrication model is employed. Wang et al. [78] demonstrated that, in deep-sea environments characterized by high pressure, low temperature, and corrosive conditions, their model exhibits significantly superior predictive accuracy for water film stiffness compared to traditional linear models, providing critical theoretical support for the design of water-lubricated gear transmissions.

### 3.3. Applicability and Challenges of EHL in Deep-Sea Environments

Under ultra-high hydrostatic pressure, conventional viscosity-pressure relationships often fail to characterize lubricant behavior accurately. As pressure approaches the glass transition point, the constitutive behavior deviates significantly from standard models [79,80]. High pressure compresses the lubricant substantially, increasing its density. This change directly impacts the accuracy of mass conservation and oil film thickness predictions in TEHL calculations [81]. The deep-sea environment creates a unique cold-exterior, hot-interior scenario. While the system is externally cooled by low-temperature seawater, the internal high-speed shearing of gear interfaces generates substantial frictional heat. This severe temperature gradient exacerbates thermal conduction and convection complexities, imposing stringent requirements for accurate prediction of oil film temperature fields and flash temperatures [82]. While intensive external cooling effectively suppresses temperature rise and preserves higher oil film viscosity, it concurrently imposes additional complexity on thermal management systems. Consequently, the TEHL modeling framework requires modified thermal boundary conditions to account for forced convection induced by seawater. The analysis must incorporate the influence of thermal management strategies, including insulation layers and active cooling systems, on the system’s thermal equilibrium [83,84]. Extremely low ambient temperatures cause a sharp increase in lubricant viscosity and reduced fluidity [85]. Although high viscosity theoretically promotes thicker oil film formation, the lubricant may fail to replenish the contact zone in time under low-speed conditions where hydrodynamic effects are weak. This can force gears into a mixed or boundary lubrication regime, significantly increasing the risk of wear [86,87]. During operation, shear heat in the meshing zone gradually raises the oil temperature, reducing viscosity and potentially shifting the lubrication state toward full-film EHL [88]. However, the deep-sea, low-temperature environment acts as a continuous cold sink, absorbing system heat and maintaining a thermal equilibrium temperature far below normal operating conditions, thereby further affecting lubricant performance and oil film formation. The combination of elevated hydrostatic pressure and Hertzian contact pressure induces exceptionally high lubricant viscosity within the conjunction zone. This pronounced viscosity amplification intensifies viscous shear stresses, consequently elevating the friction coefficient and generating substantial temperature increases. The resulting temperature rise reduces viscosity, thereby limiting the sustained growth of oil film thickness [89,90]. Since gear meshing is inherently a transient process with continuously changing contact geometry, load, and speed, extreme physical property variations in the deep sea can cause severe fluctuations in lubrication state within a single meshing cycle, making prediction and control significantly more difficult [91,92,93].

The extreme viscosity induced by high pressure substantially exacerbates the computational complexity of solving thermo-elastohydrodynamic lubrication (TEHL) governing equations, frequently precipitating convergence failures and numerical instability. Under such conditions, lubricants may manifest solid-like shear characteristics, resulting in a pronounced escalation of frictional resistance and fundamentally challenging the validity of the classical Reynolds equation, which is predicated upon continuum fluid mechanics assumptions [94,95]. Although recent research has focused on material property models for extreme environments, a model that accurately describes lubricant rheology under the simultaneous influence of low temperature, ultra-high pressure, and high shear rates is still lacking [96,97]. The interplay between time-varying frictional heat and rough interfaces with multi-scale fractal characteristics adds further complexity to the analysis of TEHL behavior [98,99,100].

## 4. Meshing Interface Texturing Technology and Its Application Prospects in Deep-Sea Gear Lubrication

### 4.1. Mechanisms of Friction Reduction and Wear Resistance in Micro-Textured Meshing Interfaces

Meshing interface texturing entails the precise design and fabrication of micro-scale and nanoscale meshing interface features, such as pits or grooves with specific geometries and spatial distributions, on friction pairs. This approach actively tailors the tribological properties of the interface [101,102,103]. By modifying meshing interface morphology, texturing regulates lubricant flow and optimizes contact stress distribution, thereby enhancing macroscopic tribological performance. Specific benefits include reduced friction coefficients, improved load-carrying capacity, minimized wear, and effective containment of wear debris [104,105]. Driven by advancements in precision manufacturing, particularly laser processing, the technique has been widely adopted in mechanical seals, bearings, and piston rings. Moreover, it holds considerable promise for mitigating lubrication challenges in heavily loaded, high-speed gear systems [106].

**Figure 9:**
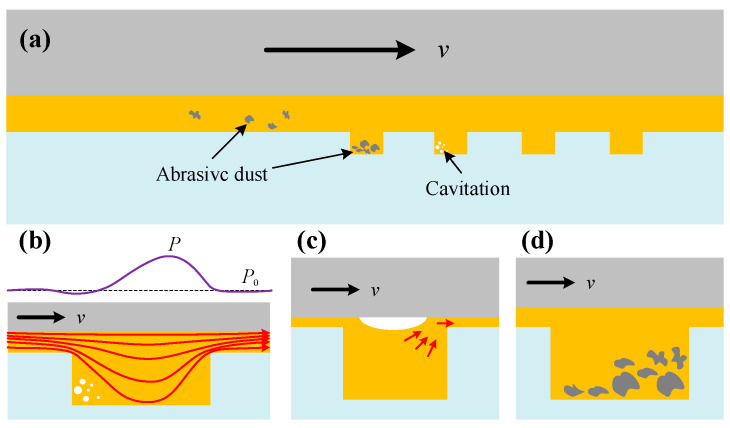
Schematic diagram of the anti-wear mechanisms of interface texturing under different operating conditions. (**a**) Debris entrapment and cavitation mechanisms on textured surfaces; (**b**) Friction and wear reduction via fluid shear replacing solid-solid contact; (**c**) Enhanced gear reliability under low-speed, heavy-load conditions; and (**d**) Wear debris trapping by interface textures to prevent three-body abrasion.

The tribological benefits of interface texturing, specifically friction reduction and wear resistance, stem from the synergy of multiple mechanisms: hydrodynamic pressure enhancement, secondary lubrication, debris entrapment, and interface strengthening [107]. Figure 9a depicts the mechanisms of debris entrapment and cavitation generation on textured interfaces. In hydrodynamic or mixed lubrication regimes, micro-textures establish microscopic wedge-shaped gaps that disrupt the parallel flow of the lubricant film, inducing a convergent zone downstream of the structures. Based on the Reynolds equation, this convergent zone generates additional hydrodynamic pressure, increasing the oil film thickness and separating the contact interfaces. By converting solid–solid contact to fluid shear, this mechanism significantly mitigates friction and wear (Figure 9b). During boundary lubrication or transient phases, such as start-stop operations, interface textures act as micro-reservoirs. Stored lubricant is released to compensate for insufficiencies in the main oil film, creating a secondary lubricating film that delays or avoids dry contact. This capability markedly improves the reliability of gears operating under severe conditions, such as low speed and heavy load (Figure 9c). Additionally, interface textures serve to trap and isolate wear debris, thereby preventing three-body abrasion at the interface (Figure 9d). High-energy beam machining techniques, such as laser texturing, induce rapid thermal cycling that modifies the microstructure surrounding the textures. This results in fine-grain strengthening and increased dislocation density, forming hardened zones with elevated microhardness that further bolster the material’s resistance to plastic deformation and wear [108].

These interface texturing mechanisms prove particularly effective in marine environments. Under high-pressure conditions, the textures’ capacity to store oil compensates for lubricant displacement, thereby maintaining the integrity of the lubricating film. In humid or water-rich environments, the geometric structure helps collect and divert water, thereby alleviating lubricant emulsification. During cold starts, the lubricant reservoir within the textures rapidly activates, forming a film that markedly reduces wear during the critical start-up period.

### 4.2. Micro-Texturing Technologies for Gear Meshing Interfaces: From Lubrication Mechanisms to Anti-Scuffing Load-Bearing Applications

**Figure 10:**
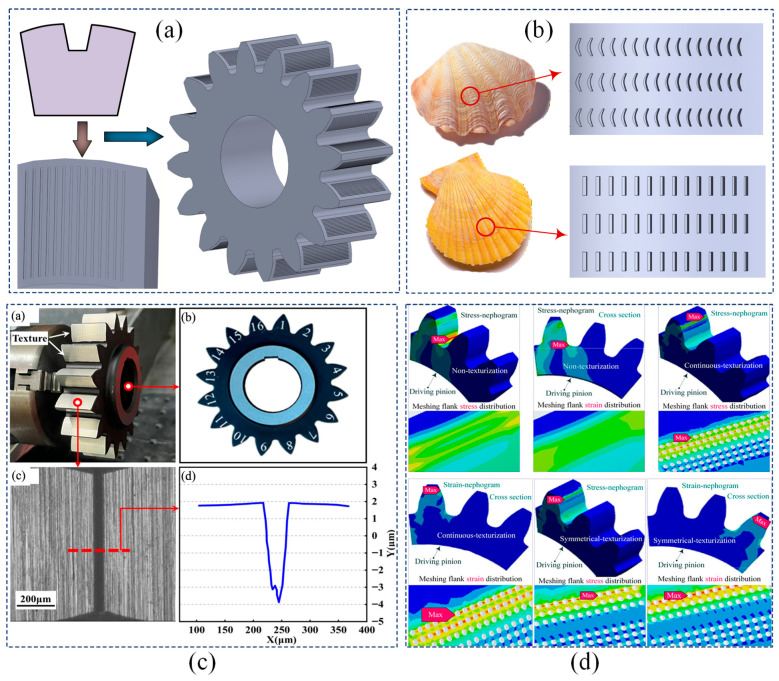
Different gear meshing interface textures. Figure (**a**) Three-dimensional model of the gear with a micro-textured meshing interface; Figure (**b**) Bionic-inspired MET configuration design; Figure (**c**) Preparation of the gear interface micro-texture, simulation model, micro-morphology, and experimental results, where in Figure (**c**): (**a**) Micro-texture processing of the gear meshing interface; (**b**) Three-dimensional simulation model; (**c**) Micro-morphology of the meshing interface; (**d**) Experimental data results. Figure (**d**) Stress field analysis of the micro-textured meshing interface. (Figure 10c: Adapted from Ref. [113], Lubricants 2025; Figure 10d: Adapted from Ref. [5], Machines 2025).

Gear interface texturing is regarded as a pivotal approach for enhancing the tribological performance of gears. Its research scope has progressively evolved from early investigations of simple geometric configurations to the deep integration of biomimetic designs and multi-parameter collaborative optimization. Preliminary explorations primarily focused on analyzing the mechanisms by which basic geometric features, such as circles and grooves, influence gear performance. Comparative studies have demonstrated that circular pits effectively reduce the friction coefficient [109,110]. Meanwhile, through parameter optimization, it has been confirmed that rationally designed groove textures can significantly decrease the interface damage rate and improve anti-adhesion capability, as illustrated in Figure 10a. Drawing inspiration from natural interface morphologies, biomimetic interface texturing has emerged as a prominent research avenue. A striped interface architecture inspired by intertidal bivalves has been developed, yielding over 20% enhancement in gear contact fatigue resistance [111]. Figure 10b shows the curved groove texture bio-inspired by Pecten maximus (great scallop), which demonstrated superior tribological performance with reduced friction coefficient, wear depth, and operating temperature [112]. The biomimetic hexagonal texture featuring bidirectional grooves is designed to enhance thermal management and prolong gear service life through improved convective heat transfer and lubricant distribution [113], as shown in Figure 10c.

To further improve performance, the research focus has shifted towards the precise optimization of key texture parameters and their coupling effects within multi-physics fields. Modeling and analysis of rhombic micro-textures indicate that these textures can reduce the friction coefficient by up to 22.96%, revealing the coupling influence of texture parameters on lubrication and vibration performance [114]. Systematic comparisons across diverse biomimetic micro-texture configurations demonstrate that crescent-shaped textures exhibit the most pronounced tribological benefits, stemming from their exceptional hydrodynamic lubrication effects and efficient wear debris accommodation [115]. The operational adaptability of these textures under varying working conditions constitutes an additional research imperative. It has been noted that micro-cylindrical pits are effective for vibration attenuation and debris entrapment under fully lubricated conditions [116,117,118]. A hybrid manufacturing technique integrating power honing with adaptive topology modification is proposed, enabling predictive active control of gear interface micro-textures for effective vibration and noise reduction [119]. As illustrated in Figure 10d, pre-designed vertical ellipsoidal pits not only enhance cooling and vibration suppression under lubrication-starved conditions but also mitigate stress concentration, optimize stress distribution, and reduce meshing strain through geometric optimization [5]. The study further elucidates the nonlinear dependence of debris capture capability and meshing stiffness on texture parameters, thereby establishing a theoretical foundation for the optimal design of interface textures under complex operating conditions [120].

The fabrication of high-performance textures relies on manufacturing technologies characterized by high precision, high efficiency, and low damage. Current mainstream processes are categorized into subtractive, additive, and material-transfer types, with subtractive manufacturing being the most widely used. Among subtractive techniques, laser processing, particularly femtosecond laser processing, has emerged as the preferred method due to its high precision and flexibility. By adjusting parameters such as laser power and scanning speed, texture morphology can be precisely controlled and meshing interface hardness enhanced; however, challenges regarding the heat-affected zone and slag must be addressed [121,122]. Chemical etching uses etchants to remove material directionally; it is suitable for mass production and preserves the mechanical properties of the substrate, yet is limited by corrosiveness and processing speed [123,124,125]. Micro-grinding enables superior interface quality and the fabrication of complex micro-grooves, whereas abrasive flow machining offers excellent uniformity on complex gear meshing interfaces but presents challenges in shape control [126,127]. Abrasive water jet machining removes material via high-speed abrasive impingement, offering potential for high efficiency and environmental friendliness [128,129,130]. Conversely, electrical discharge machining, while suitable for hard conductive materials, is associated with issues such as recast layers and low efficiency. Regarding additive techniques, 3D printing (additive manufacturing) can fabricate complex three-dimensional structures but currently faces limitations related to material grain structure and cracking [131,132]. Material transfer techniques, such as micro-texture rolling, leverage plastic deformation to transfer patterns, offering high efficiency and consistency; however, challenges exist when processing high-hardness or complex textures [133]. A process combining laser pre-treatment with abrasive flow machining is adopted, which significantly enhanced the quality and wear resistance of the gear meshing interfaces [134,135]. Currently, process selection requires careful consideration of material characteristics and costs. Looking ahead, driven by the development of hybrid processes (e.g., laser-electrochemical combinations, integration of abrasive water jet with milling) and digital control technologies, the manufacturing of gear interface textures is poised to evolve towards greater efficiency and precision.

### 4.3. Meshing Interface Enriched Lubrication Considering Micro-Texture Effects

For accurate prediction and design of lubrication performance in textured gears, theoretical research has progressed from simplified isothermal models to sophisticated multi-physics simulations [136]. Early investigations predominantly relied upon isothermal assumptions, with particular emphasis on elucidating the effects of interface texture parameters on lubrication characteristics [137]. It has been experimentally validated that increasing the micro-dimple area ratio contributes to enhanced oil film thickness. The beneficial effects of groove-type textures and laser interface texturing on hydrodynamic performance have been confirmed. Transient EHL theory is utilized to investigate the effects of composite curvature radius, entrainment velocity, and applied load on lubrication regime transitions. The influence of texture orientation is examined. These findings provide a theoretical foundation for the optimization of geometric texture parameters [138,139,140].

As the understanding of lubrication mechanisms has deepened, research has increasingly focused on complex interfacial behaviors and thermo-dynamic coupling effects. The patterns by which interface texture-induced cavitation effects vary with rotational speed, load, and geometric parameters are revealed [141]. A multiphysics coupling model incorporating interface roughness and micro-texture topography has been developed, revealing that aspect ratio optimization can achieve approximately 50% reduction in contact stress at gear meshing interfaces, concomitantly enhancing fatigue durability [142]. Considering the significant thermomechanical coupling effects prevalent under high-speed, heavy-load operating conditions, research emphasis has progressively migrated toward TEHL and dynamic-coupling modeling frameworks. A three-dimensional thermoelastic-dynamic contact model is proposed [143], and mixed lubrication analysis coupled with interfacial friction-dynamics modeling is employed to elucidate the intricate effects of interface textures on contact stiffness, damping, and system vibrations [144,145]. The results demonstrate that the interplay between interfacial roughness and texture patterns is directly correlated with the dynamic stability and thermal characteristics of gear systems.

In light of the complexity inherent in the continuous dynamic process of gear meshing, research trends are shifting from single-point analysis towards multi-scale coupling and system-level simulation. On one hand, multi-scale coupling analysis of micro-textured meshing interfaces has emerged as a prominent research area. This approach embeds local lubrication boundaries that account for texture effects into macroscopic dynamic models, thereby enabling precise mapping between microstructural features and macroscopic motion. On the other hand, research focuses on system-level evaluation, comprehensively considering the influence of textured gear pairs on the transmission’s overall efficiency, vibration, noise, and temperature rise. These frontier directions not only deepen the understanding of the underlying mechanisms of textures but also provide critical support for the global optimization of high-performance gear transmission systems; however, their implementation also imposes higher demands on computational capabilities and numerical algorithms.

### 4.4. Multiscale Micro-Texturing Meshing Interface for Deep-Sea Gear Transmission Environmental Challenge

In recent years, numerous scholars have conducted experimental investigations into the tribological performance of interface textures in marine environments. These studies typically employ experimental setups that simulate marine conditions, such as seawater-lubricated bearing test rigs and high-pressure chamber friction testers, to validate the efficacy of interface textures. A study focusing on marine propeller hub bearings demonstrated that interface texturing technology can significantly enhance tribological performance, reducing the friction coefficient by 20–30% and the wear volume by 40–50%. This improvement is particularly pronounced under starved lubrication conditions, as the textures provide a continuous lubricant supply, thereby reducing the likelihood of direct metal-to-metal contact. Another investigation into the tribological properties of textured titanium alloys under dry friction and perfluoropolyether oil lubrication found that appropriately designed interface textures can significantly improve the wear resistance of titanium alloys, especially in harsh environments. This is of great significance for titanium alloy gears, which are widely utilized in marine equipment. Research has explored the tribological performance of a combined system involving interface texturing and ionic liquid lubrication in marine settings. The results indicated that these factors exhibit a synergistic effect, which is capable of further enhancing the wear and corrosion resistance of gears. This provides novel insights for the development of specialized gear lubrication systems tailored for marine environments.

Although interface and texturing technology have demonstrated promising results in laboratory studies, its practical application in marine gear transmission systems faces several challenges, including high fabrication costs, long-term durability, and the impact on gear strength. These issues require further investigation and resolution to facilitate the engineering application of this technology. While current research on gear meshing interface texturing specifically for marine environments is limited, analysis of the underlying mechanisms suggests that interface textures hold immense potential for addressing marine environmental challenges. The oil storage capacity of textures can supply essential lubrication to the meshing zone during the startup phase, characterized by low temperatures, high viscosity, and poor lubricant fluidity, thereby preventing dry friction. When lubricant viscosity decreases due to seawater contamination, the secondary hydrodynamic effect generated by textures can partially compensate for the loss of load-carrying capacity, helping to maintain the necessary oil film thickness. While interface textures themselves do not possess intrinsic corrosion resistance, they provide a physical carrier for integrating anti-corrosion and friction-reduction technologies. Conversely, a purely textured interface may even accelerate localized corrosion due to the increased interface area and the formation of occluded cells. Therefore, it is essential to consider interface texturing and anti-corrosion coating technologies in synergy.

## 5. Conclusions and Prospects

This study presents a systematic review of how the distinctive and harsh marine environment, characterized by extreme hydrostatic pressure and cryogenic temperatures, influences the lubrication performance of gear transmission systems. Centered on EHL theory and interface micro-texturing technology, it synthesizes recent research advances, identifies critical scientific gaps, and outlines key technical challenges in deep-sea tribological applications.

The marine environment, characterized by high pressure and low temperature, poses multiple challenges for gear lubrication by significantly altering the viscosity-pressure, viscosity-temperature, density, and thermophysical properties of lubricants. The ultra-high viscosity resulting from the coupling of high pressure and low temperature is a core issue, leading to problems such as poor fluidity, difficult startup, high churning losses, and lubricant starvation. The risks of emulsification, corrosion, and additive failure associated with seawater intrusion further exacerbate the likelihood of lubrication failure.

EHL theory has effectively guided the design of gear lubrication systems. However, the drastic temperature and pressure changes in extreme deep-sea environments render the viscosity-pressure characteristics, rheological behavior, and thermal effects of lubricants non-negligible, prompting the evolution of gear EHL theory towards TEHL. Although current TEHL research has established transient numerical models coupling thermal effects, non-Newtonian rheology, and realistic interface topography, simulation studies specifically targeting deep-sea high-pressure and low-temperature environments remain limited. The primary bottlenecks are the lack of lubricant property data under extreme operating conditions and numerical convergence challenges arising from multi-field coupling.

Interface texture technology effectively enhances the storage, flow, and load-carrying capacity of lubricants by fabricating microstructures on the gear interface, thereby reducing gear friction and wear. Optimal interface texture design can significantly improve the tribological performance of gears under mixed and boundary lubrication conditions, making it theoretically suitable for addressing challenges such as low-temperature startup and low-speed heavy-load operations in deep-sea gears. However, to fully harness the potential of interface textures, it is necessary to optimize their parameters for specific operating conditions and address long-term reliability issues in deep-sea environments.

Despite significant progress in research on deep-sea gear transmission lubrication, existing theories and technologies still face numerous bottlenecks under the combined challenges of extremely high pressure, low temperature, and severe corrosion, necessitating further in-depth exploration.

High pressure and low temperature significantly alter the physical properties of lubricants. At the same time, seawater corrosion fundamentally undermines both lubricant performance and gear meshing interface integrity, posing a risk of failure to traditional TEHL theory. Currently, detailed mechanistic studies on how seawater corrosion affects the TEHL oil film thickness, pressure distribution, and temperature fields are scarce. Furthermore, there is a lack of systematic understanding regarding the evolutionary patterns of gear material wear and lubricant aging under multi-field coupling. Theoretical research on synergistic design, optimization methods, and case studies of gear interface textures and anti-corrosion coatings is limited, with applications in high-pressure, low-temperature marine environments still in their infancy. Additionally, laboratory simulations struggle to fully replicate actual deep-sea operating conditions, meaning the long-term durability of integrated texture-coating interfaces and their impact on system dynamic characteristics require further verification. To date, no comprehensive literature exists that integrates TEHL, interface textures, anti-corrosion coatings, and specialty lubricants to provide a complete technical framework for addressing these issues.

As marine equipment evolves toward greater depths and extended mission endurance, future research should focus on developing multi-physics coupling simulation models to achieve precise prediction and optimization of the corrosion-coating-texture-lubrication system. It is essential to establish high-fidelity experimental testing platforms, integrated with in situ long-term monitoring and real-time condition sensing technologies, to provide reliable data support for the development and validation of novel materials and innovative designs. Concurrently, by integrating multidisciplinary technologies from materials science, fluid mechanics, and data science, the engineering application of lubricants and integrated texture-coating manufacturing processes should be promoted, ultimately achieving high reliability and long service life for gear transmission systems in marine environments. Future research will integrate high-pressure rheological experiments with Molecular Dynamics (MD) simulations to establish a collaborative optimization framework for lubricant formulation and micro-texture geometry under hydrostatic compression conditions, thereby supporting the full-life-cycle reliability design of deep-sea gear transmission systems.

## Figures and Tables

**Table 1:** Parameters related to lubrication media for deep-sea gear transmission system.

Type	Model	Formula	Equation Number
Viscosity–temperature correlation	Reynolds	$\eta =\eta_{0}e^{-\beta \left(T-T_{0}\right)}$	(1)
	Andrade-Erying	$\eta =\eta_{0}e^{\frac{\alpha }{T}}$	(2)
	Slotte	$\eta =\frac{s}{{\left(\alpha +T\right)}^{m}}$	(3)
	Vogel	$\eta =\eta_{0}e^{b/\left(T+\theta \right)}$	(4)
	Walther	$\left(v+a\right)=bd^{1/T^{c}}$	(5)
Viscosity–pressure correlation	Barus	$\eta =\eta_{0}e^{\alpha p}$	(6)
	Roelands	$\eta =\eta_{0}e^{\left\{\left(\ln\eta_{0}+9.67\right)\left[-1+{\left(1+p_{0}p\right)}^{z}\right]\right\}}$	(7)
	Cameron	$\eta =\eta_{0}{\left(1+cp\right)}^{16}$	(8)
Viscosity–temperature–pressure correlation	Barus and Reynolds	$\eta =\eta_{0}e^{\left[\alpha p-\beta \left(T-T_{0}\right)\right]}$	(9)
	Roelands	$\eta =\eta_{0}e^{\left\{\left(\ln\eta_{0}+9.67\right)\left[{\left(1+5.1\times 10^{-9}p\right)}^{0.69}\times {\left(\frac{T-138}{T_{0}-138}\right)}^{-1.1}-1\right]\right\}}$	(10)
	WLF-Yasutom	$\eta =\eta_{g}e^{\left(\log\left(10\right)\frac{-C_{1}\left(T-T_{g}\left(p\right)\right)F\left(p\right)}{C_{2}+\left(T-T_{g}\left(p\right)\right)F\left(p\right)}\right)}$	(11)

**Table 2:** Dynamic viscosity of lubricating oil under different temperatures and pressures.

Temperature	5 MPa	15 MPa	20 MPa	25 MPa	30 MPa
0 °C	5.2584	6.6763	7.4744	8.1742	8.7312
5 °C	4.2642	5.5105	6.2095	6.8115	7.3204
10 °C	3.2655	4.2734	4.8230	5.3308	5.7945

## Data Availability

No new data were created or analyzed in this study. Data sharing is not applicable to this article.
